# Allostatic Load Predicts Immune-Related Toxicity and Survival in Melanoma Patients Receiving Immune Checkpoint Inhibitors

**DOI:** 10.3390/cancers18040606

**Published:** 2026-02-12

**Authors:** Jie Shen, Yufan Guan, Chase Myers, Roger T. Anderson, Elizabeth M. Gaughan, Hua Zhao

**Affiliations:** 1 Department of Public Health Sciences, School of Medicine, University of Virginia, Charlottesville, VA 22903, USA; 2 Division of Hematology & Oncology, Department of Medicine, School of Medicine, University of Virginia, Charlottesville, VA 22903, USA

**Keywords:** allostatic load, immunotherapy, melanoma, outcomes

## Abstract

Background: Host physiological factors may influence immune response, treatment tolerance, and survival during immune checkpoint inhibitor (ICI) therapy. Allostatic load (AL) summarizes cumulative physiological dysregulation across multiple biological systems. We evaluated whether pre-treatment AL is associated with immune-related toxicity and clinical outcomes among patients with advanced melanoma receiving ICIs. Methods: We analyzed 399 patients with melanoma treated with ICIs at the University of Virginia Cancer Center (2013–2025). AL was derived from routinely collected clinical laboratory biomarkers measured prior to treatment initiation. Multinominal logistic and Cox regression models assessed associations between AL and immune-related adverse events (irAEs), treatment response, disease progression, and overall survival (OS), adjusting for demographic, clinical, and treatment factors. Results: The mean AL score was 4.43. Each 1-unit increase in AL was associated with higher odds of grade ≥ 2 toxicity (adjusted odds ratio [OR] = 1.30; 95% confidence interval [CI]: 1.08–1.57). Among patients who developed irAEs, higher AL was associated with poorer treatment response (adjusted OR = 1.24; 95% CI: 1.01–1.54) and increased risk of disease progression (adjusted hazard ratio [HR] = 1.14; 95% CI: 0.98–1.33). Higher AL was also associated with shorter OS, with a 26% higher mortality risk per 1-unit increase in AL (adjusted HR = 1.26; 95% CI: 1.14–1.39). Conclusions: Higher pre-treatment AL was associated with increased immune-related toxicity and poorer survival in melanoma patients treated with ICIs. AL represents a feasible pre-treatment marker of host physiological vulnerability that may complement existing clinical predictors. Prospective studies are needed to validate these findings and assess clinical utility.

## Simple Summary

Immune checkpoint inhibitors improve survival in melanoma, but patients differ in their ability to tolerate treatment and benefit from therapy. We examined whether allostatic load, a measure of cumulative physiological stress derived from routine clinical tests, was associated with outcomes in melanoma patients receiving immunotherapy. Patients with higher allostatic load before treatment were more likely to develop immune-related side effects and had poorer survival. These findings suggest that baseline physiological vulnerability may influence immunotherapy outcomes and could help identify patients who need closer monitoring.

## 1. Introduction

Immune checkpoint inhibitors (ICIs) have transformed the treatment landscape for advanced melanoma, producing durable responses and long-term survival in a substantial proportion of patients [1,2,3]. However, clinical outcomes remain highly heterogeneous. A significant fraction of patients experience immune-related adverse events (irAEs), treatment discontinuation, or disease progression [3,4,5,6], and reliable tools to identify patients at elevated risk for toxicity or poor outcomes before treatment initiation remain limited [7,8,9]. Current predictive efforts have focused largely on tumor-intrinsic and immunogenomic features, such as tumor mutational burden, PD-L1 expression, and tumor microenvironment characteristics, which incompletely account for variability in treatment tolerance and survival [10,11].

Increasing evidence suggests that host-level physiological factors play an important role in shaping immunotherapy outcomes. Aging, comorbid conditions, metabolic dysfunction, systemic inflammation, and psychosocial stress have all been associated with altered immune regulation and differential responses to ICIs [10,11,12]. Inflammatory markers, such as the neutrophil-to-lymphocyte ratio and the Glasgow Prognostic Score, as well as comorbidity indices, have demonstrated prognostic value in ICI-treated populations [13,14,15]. While clinically useful, these measures typically capture single domains of host biology and may not fully reflect cumulative physiological vulnerability.

Allostatic load (AL) is a composite measure developed to quantify the cumulative burden of chronic physiological stress across multiple biological systems [16,17,18]. Rather than indexing acute stress responses, AL reflects long-term dysregulation in metabolic, cardiovascular, and inflammatory pathways that accumulate over time and are shaped by aging, chronic disease, and environmental exposures. Higher AL has been associated with frailty, functional decline, multimorbidity, and mortality in population-based studies [16,19,20], and emerging evidence links AL to cancer risk and survival [20,21,22,23]. However, the relevance of AL to cancer immunotherapy outcomes remains largely unexplored.

Conceptually, AL offers a framework to capture host physiological resilience which may be especially relevant in the context of ICIs, which rely on immune activation and can precipitate systemic inflammatory and autoimmune toxicities [24,25]. Patients with greater baseline physiological dysregulation may be less able to tolerate immune perturbation or recover from treatment-related injury, potentially influencing both toxicity risk and survival. Importantly, AL can be operationalized using standard clinical laboratory measures, making it feasible to assess in real-world oncology settings without specialized assays.

Despite these theoretical considerations, several critical gaps remain. Prior studies have not systematically evaluated whether pre-treatment AL is associated with irAE risk, severity, or survival in patients receiving ICIs for melanoma. Moreover, it is unclear whether AL provides information that complements established clinical prognostic factors or whether its influence on outcomes may differ depending on the occurrence of immune-related toxicity. Addressing these gaps is necessary to clarify the potential role of AL as a host-level biomarker of vulnerability in immuno-oncology. To address this gap, we examined the association between pre-treatment AL and subsequent toxicity, treatment response, disease progression, and overall survival among 399 advanced melanoma patients treated with ICIs at the University of Virginia Comprehensive Cancer Center from 2013 to 2025. We hypothesized that higher AL would be associated with (1) increased incidence and severity of irAEs, (2) poorer treatment response, and (3) shorter overall survival.

## 2. Methods

### 2.1. Study Population

A total of 399 melanoma patients were identified from the melanoma patient pool at the University of Virginia Comprehensive Cancer Center between 2013 and 2025. Eligible participants met the following criteria: (1) histologically confirmed advanced cutaneous melanoma; (2) receipt of at least one immune checkpoint inhibitor (ICI)—including anti-PD-1, anti-CTLA-4, or combination therapy—during the study period; (3) age ≥ 18 years at the time of treatment; (4) availability of sufficient pre-treatment clinical and laboratory data to construct the AL score; and (5) active clinical follow-up after treatment initiation. The study followed the Strengthening the Reporting of Observational Studies in Epidemiology (STROBE) guidelines for study design, data analysis, and interpretation. The protocol (#301091, approved 9 October 2023) was reviewed and approved by the Institutional Review Board (IRB) of the University of Virginia, which granted a waiver of informed consent due to the retrospective nature of the analysis.

### 2.2. AL Construction

In this study, 14 biomarkers were selected based on their routine availability in clinical settings and established relevance to stress-related pathophysiology [21,26]. The biomarker selection was guided by established AL frameworks that emphasize multisystem physiological dysregulation across cardiovascular, metabolic, and inflammatory domains, while prioritizing markers that are routinely collected in oncology practice and available at standardized pre-treatment time points in our cohort. These biomarkers captured four physiological domains: cardiovascular (heart rate, systolic and diastolic blood pressure), metabolic (body mass index [BMI], triglycerides, high-density lipoprotein cholesterol [HDL-C], low-density lipoprotein cholesterol [LDL-C], total cholesterol [TC], alkaline phosphatase [ALP], fasting glucose, albumin), renal (creatinine, estimated glomerular filtration rate [eGFR], blood urea nitrogen [BUN]), and immune function (white blood cell [WBC] count). Missing data were handled using multiple imputation via chained equations (MICE) [27], generating 10 imputed datasets. Continuous variables were imputed using predictive mean matching, and parameter estimates and standard errors were pooled according to Rubin’s rules. Each biomarker was dichotomized as high-risk or low-risk based on established clinical guidelines (Table S1). The cumulative AL score was then calculated as the sum of all high-risk markers, ranging from 0 to 14. For analytical purposes, AL was dichotomized into low and high categories by median level (AL = 4), corresponding to the distribution of AL scores within the study population.

### 2.3. Assessment of Covariates and Study Outcomes

Covariates included demographic variables (age at first drug administration and sex) and clinical treatment characteristics (tumor stage, mutation status, brain metastases, prior receipt of adjuvant therapy, and therapy drug class). All covariates were considered potential confounders. Mutation status was categorized as “*BRAF*−”, “*BRAF+*”, or “Other”. Brain metastases and adjuvant therapy were coded as “yes” or “no”. Therapy drug class was defined as CTLA-4 plus PD-1 combination therapy or PD-1 monotherapy. The primary outcome was treatment-related toxicity. Secondary outcomes included treatment response, disease progression and overall survival. IrAEs in this study were identified based on real-time clinical documentation by the treating oncology clinicians, who routinely evaluate and manage immunotherapy-related toxicities as part of standard care. Events were recorded contemporaneously in the medical record at the time of occurrence, rather than retrospectively inferred from administrative codes alone. Each adverse event was graded on a scale from 1 to 4 based on CTCAE. Treatment response was categorized as complete response (CR), partial response (PR), stable disease (SD), or progressive disease (PD). Disease progression was defined as the time from the date of first ICI administration to the date of first documented progression. Overall survival was defined as the time from the date of first ICI administration to death from any cause. Participants who remained alive at the end of the study period were censored at the date of last known contact or at study completion, whichever occurred first.

### 2.4. Statistical Analysis

Descriptive statistics were used to summarize the distribution of demographic and clinical treatment characteristics within the study population. Mean differences in AL were compared across key characteristics using Students’ test (for two categories) or ANOVA (>2 categories). Multinomial logistic regression models were applied to evaluate the associations between AL and treatment-related toxicity grades, as well as treatment response categories, with results expressed as odds ratios (ORs) and 95% confidence intervals (CIs). Time-to-event outcomes, including disease progression and overall survival (OS), were analyzed using Cox proportional hazards models, and results were reported as hazard ratios (HRs) with 95% CIs. For both multinomial logistics and Cox proportional hazards models, three hierarchical models were constructed to assess the robustness of associations: Model 1 adjusted for age and sex; Model 2 additionally adjusted for tumor stage, *BRAF* mutation, and brain metastases; and Model 3 further adjusted for, adjuvant therapy, and therapy drug class. For progression and survival analyses, toxicity status was also included in Models 2–3. The proportional hazards assumption was evaluated using Schoenfeld residuals, with no violations detected. To assess the discriminative performance of the survival models, we calculated Harrell’s concordance index (C-index). We compared model discrimination with and without AL to quantify the incremental predictive value of AL. A paired bootstrap test (1000 resamples) was applied to assess the improvement of C-index. To assess the robustness of AL construction, we carried out sensitivity analyses using alternative AL constructions, including, a quartile-based AL formulation and a reduced core multisystem AL score. All statistical tests were two-sided, and *p* < 0.05 was considered statistically significant. Analyses were performed using R software, version 4.3.0.

## 3. Results

A total of 399 patients with advanced melanoma who received ICI therapy were included in the analytic cohort (Table 1). The mean pre-treatment AL score was 4.43 ± 2.13, ranging from 0 to 11. The mean (SD) age at treatment initiation was 64.0 ± 13.6 years, and 63.9% of patients were male. The cohort was predominantly White (96.7%), consistent with regional melanoma demographics. Most patients (89.2%) had stage IV disease at the start of immunotherapy, while 10.8% had stage III disease. Approximately 15.3% had brain metastases at baseline. *BRAF* mutations were present in 34.6% of tested patients. Regarding treatment regimen, nivolumab and pembrolizumab were the most common monotherapies (37.6% and 33.1%, respectively), followed by ipilimumab + nivolumab combination therapy (21.6%) and ipilimumab monotherapy (7.7%). AL scores were significantly higher among older patients (>64 years) compared with younger patients (mean = 4.73 vs. 4.13, *p* = 0.04) and were modestly higher among males than females (*p* = 0.05). No significant differences in AL were observed by race, BRAF mutation status, disease stage, brain metastases, or immunotherapy regimen (all *p* > 0.10).

**Figure 1:**
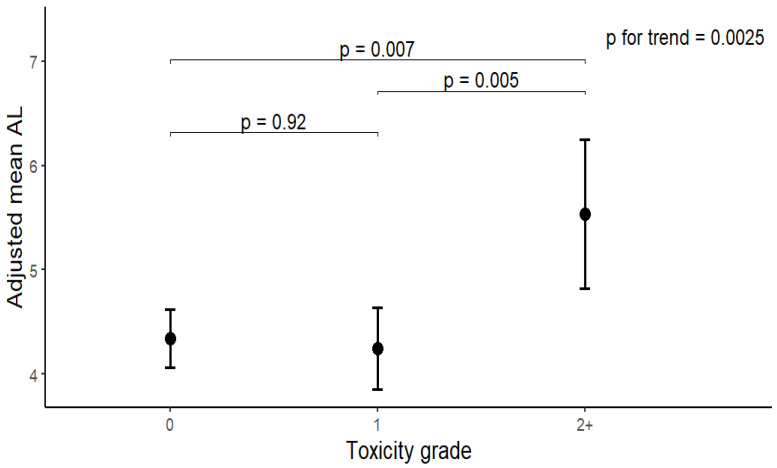
Levels of pre-treatment AL by toxicity grade among melanoma patients treated with immunotherapy.

Overall, 43.9% of patients developed irAEs, and 8% experienced grade ≥ 2 toxicities (Table 1). As shown in Figure 1, pre-treatment AL rose progressively with increasing toxicity grade. Pre-treatment AL was highest among those who later developed severe (grade ≥ 2) irAEs (mean = 5.4) compared with those with no (4.4) or mild (grade 1, 4.2) events (*p* = 0.007 and 0.005, respectively). In multinomial logistic regression analyses, each 1-unit increase in AL was associated with a 21% increased risk of developing grade ≥ 2 toxicity in the crude model (OR = 1.21, 95% CI: 1.03, 1.43) (Table 2). After sequential adjustment for demographic, clinical, and treatment variables, the association remained significant. Each 1-unit increase in AL corresponded to a 27–30% higher odds of developing grade ≥ 2 toxicity (Model 1: OR = 1.29, 95% CI: 1.09, 1.53; Model 2: OR = 1.27, 95% CI: 1.07, 1.52; Model 3: OR = 1.30, 95% CI: 1.08, 1.57). No significant association was found for grade 1 toxicities. The significant associations were further confirmed in sensitivities analyses using alternative AL constructions (Table S2).

When toxicity events were categorized by affected organ system (Table S3), the highest pre-treatment AL levels were observed for cardiovascular (mean 6.17), neurologic (6.25), and constitutional (6.50) toxicities, followed by hematologic (5.25) and ocular (5.33) systems. When ranked by clinical severity (Table S4), mean AL was highest among patients with severe adverse events (5.59 ± 2.27, *p* < 0.01) compared with moderate or mild categories.

Among patients with available response data (n = 325), 17.53% achieved a CR, 29.84% a PR, 20.62% SD, and 32.00% PD at best response (Table 1). Levels of pre-treatment AL did not differ significantly across response categories (*p* = 0.18) (Table 3). In multinomial logistic regression analyses, pre-treatment AL was not significantly associated with response category in either crude or adjusted models. However, a significant interaction was observed between AL and toxicity (*p* for interaction < 0.001). When stratified by toxicity status, higher AL predicted poorer treatment response among patients who experienced toxicity. In this subgroup, patients with higher AL were more likely to have non-response (SD/PD) outcomes compared with CR/PR (adjusted OR = 1.24, 95% CI: 1.01, 1.54), whereas no association was observed among patients without toxicity. Additionally, higher AL was associated with a 38% increased risk of SD compared with CR (adjusted OR = 1.38, 95% CI: 1.01, 1.89).

The median follow-up duration was 31 months (IQR = 14–55 months). Overall, 150 patients (37.6%) experienced disease progression during follow-up (Table 1). Pre-treatment AL levels did not differ between patients with and without progression (*p* = 0.46). However, in Cox regression analyses, higher pre-treatment AL was associated with a 12% increased hazard of disease progression after adjusting for demographic, clinical, and treatment factors (Model 3: HR = 1.12, 95% CI: 1.01, 1.24) (Table 4), with stronger effects observed among those who experienced toxicity (HR = 1.14, 95% CI: 0.98, 1.33).

**Figure 2:**
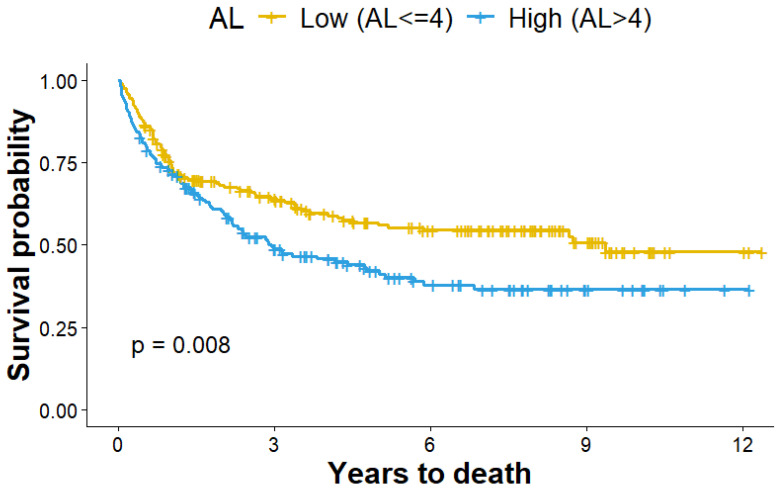
Kaplan–Meier curve: association between pre-treatment AL and overall survival among melanoma patients treated with immunotherapy.

More than half of patients (51.1%) died during follow-up (Table 1). Those who died had significantly higher pre-treatment AL scores compared with survivors (mean = 4.87 vs. 4.03, *p* < 0.001). This association was confirmed in survival analyses, where higher AL (AL > 4) was independently associated with worse OS. Kaplan–Meier curves (Figure 2) demonstrated that patients with higher pre-treatment AL (AL > 4) had significantly shorter survival compared with those with lower AL (AL ≤ 4) (log-rank *p* = 0.008). In Cox regression analysis (Table 4), higher AL was associated with worse OS in both crude and adjusted models (*p* < 0.05). In the fully adjusted model, each 1-unit increase in AL corresponded to a 26% higher hazard of death (HR = 1.26, 95% CI: 1.14, 1.39). This association remained consistent among patients with and without toxicity (HR = 1.23, 95% CI: 1.10, 1.37 and HR = 1.19, 95% CI: 0.99, 1.42, respectively). In stratified analyses among patients who experienced toxicity (Table S5), the association between AL and OS was strongest in those with documented disease progression (HR = 1.26, 95% CI: 1.01, 1.58), but not in patients without progression. Associations did not differ materially by *BRAF* mutation status (*p* for interaction > 0.1).

Finally, in the overall study population, the fully adjusted survival model without AL achieved a C-index of 0.76. When AL was included in the model, the C-index increased to 0.80 (C-Index difference = 0.04). In parried bootstrap test, the difference in C-index between two models was 0.038 (95% CI: 0.003, 0.006, *p* = 0.024), indicating that inclusion of AL statistically significantly improves the model’s ability to discriminate and supports the incremental predictive value of AL.

## 4. Discussion

In this study of patients with melanoma treated with ICIs, higher pre-treatment AL was associated with an increased risk and severity of irAEs and with poorer overall survival. Patients with elevated AL were more likely to experience grade ≥ 2 toxicities, and those with the highest AL prior to treatment initiation had the worst survival outcomes. In exploratory analyses, AL also varied across categories of organ-specific toxicity, with higher values observed among patients who developed cardiovascular, neurologic, and pulmonary events; however, these findings were based on small subgroup sizes and should be interpreted as descriptive and hypothesis-generating. Notably, the association between AL and treatment response, disease progression, and survival appeared to be modified by the occurrence of immune-related toxicity, such that adverse outcomes associated with high AL were primarily observed among patients who developed toxicity. Collectively, these findings suggest that cumulative physiological dysregulation prior to treatment initiation identifies a subset of patients who are more vulnerable to adverse clinical trajectories during immunotherapy.

Although the role of host physiological resilience and stress-related biology in cancer outcomes is well recognized [20,28], this study extends existing literature by operationalizing these concepts into a pragmatic, pre-treatment biomarker framework using routinely available clinical laboratory data in the setting of melanoma immunotherapy. Prior studies of ICI outcomes have largely focused on tumor-intrinsic or immunogenomic predictors, including tumor mutational burden, PD-L1 expression, and microbiome composition [10,11]. In contrast, patient-level factors such as comorbidities, systemic inflammation, aging, and psychosocial stress, which are known to influence immune function, have been less frequently integrated into predictive models [12]. AL provides a summary measure of multisystem physiological dysregulation spanning metabolic, cardiovascular, and inflammatory domains, thereby capturing broader host vulnerability rather than a single biological pathway.

A substantial body of literature has demonstrated that simpler systemic inflammation markers, including the neutrophil-to-lymphocyte ratio and the Glasgow Prognostic Score, as well as comorbidity indices, are associated with outcomes in ICI-treated patients [13,14,15]. These measures capture important aspects of inflammatory burden and baseline health status and remain clinically useful because of their simplicity. AL differs conceptually by integrating multiple physiological systems and reflecting cumulative dysregulation rather than acute inflammation alone. Direct comparison with these established indices was not feasible in the present study due to data availability and sample size constraints; therefore, the current findings should not be interpreted as demonstrating superiority of AL. Rather, future studies in larger, prospectively characterized cohorts should evaluate the relative and combined prognostic utility of AL and simpler inflammatory or comorbidity-based scores using complementary performance metrics beyond discrimination alone.

The observed association between AL and clinical outcomes likely reflects downstream physiological consequences of chronic stress-related dysregulation rather than direct measurement of upstream neuroendocrine pathways. Although AL is conceptually grounded in stress biology, the biomarkers included in this study do not directly assess sympathetic nervous system activity, hypothalamic–pituitary–adrenal axis dynamics, or glucocorticoid signaling. Instead, AL captures metabolic, cardiovascular, and inflammatory manifestations that may be influenced by these upstream processes. Accordingly, mechanistic interpretations linking AL to specific neuroendocrine pathways should be viewed as indirect and hypothesis-generating. Additional biomarker domains, such as cortisol dynamics, catecholamines, cytokine-level inflammatory markers, and immune cell-based metrics, were not available in this cohort and may provide complementary insight into the biological pathways linking host physiology to immunotherapy tolerance and outcomes [28,29,30]. Future prospective studies integrating these measures alongside AL will be important to refine mechanistic understanding.

The finding that the adverse prognostic impact of high AL was most evident among patients who experienced immune-related toxicity warrants careful interpretation. This pattern suggests that AL may identify a state of reduced physiological reserve or impaired regulatory capacity that becomes clinically consequential once immune perturbation occurs. However, the observational design precludes causal inference, and these results do not establish that AL modifies immune activation or treatment efficacy directly. Furthermore, patients with high AL may be more likely to experience treatment interruption or require immunosuppressive management for toxicity, which could contribute to poorer outcomes independent of underlying tumor response. These interrelated pathways cannot be disentangled in the present study and should be explored in future work.

From a predictive standpoint, inclusion of AL resulted in a modest improvement in survival discrimination (C-index increase from 0.76 to 0.80). Given that the baseline model already included strong prognostic factors such as disease stage and brain metastases, large gains in discrimination are not expected, and the observed increment should be interpreted cautiously. Importantly, we did not evaluate clinical utility using decision-analytic approaches such as decision curve analysis, nor did we benchmark AL against other parsimonious clinical risk scores. As such, claims regarding clinical meaningfulness should be limited to feasibility and potential relevance rather than established utility. Future studies with larger sample sizes and sufficient event counts should assess calibration, net benefit, and reclassification to determine whether AL meaningfully improves clinical decision-making.

Several limitations merit consideration. AL was derived from available clinical laboratory measures, which, although routinely collected and clinically interpretable, may be missing or inconsistently measured in retrospective cohorts. Certain clinically relevant factors, such as performance status (e.g., ECOG), pre-existing autoimmune conditions, and baseline corticosteroid use, which may influence both allostatic load and ICI outcomes, were not included in the analysis because these data were either largely missing or not systematically documented for all patients. The modest sample size limited power for subgroup analyses and increased uncertainty around effect size estimates, particularly for organ-specific toxicity categories. The observational design precludes causal inference, and unmeasured factors such as medication use, lifestyle behaviors, or social stressors may contribute to elevated AL. In addition, the study spanned a prolonged treatment period during which immunotherapy practices and toxicity management evolved; residual confounding by treatment era cannot be excluded. Finally, the cohort was racially homogeneous, limiting assessment of whether AL contributes to racial or socioeconomic disparities in immunotherapy outcomes.

Despite these limitations, this study supports AL as a feasible pre-treatment marker of host physiological vulnerability that is associated with immunotherapy toxicity and survival in melanoma. By capturing cumulative multisystem dysregulation using routinely available data, AL offers a complementary perspective to tumor-centric models of immunotherapy response. However, its role should be viewed as risk stratification rather than risk modification, and its clinical utility remains to be established. Prospective validation, formal comparison with simpler biomarkers, and decision-analytic evaluation will be essential next steps before AL can be considered for implementation in immunotherapy care pathways.

## 5. Conclusions

Higher pre-treatment allostatic load was associated with increased immune-related toxicity and poorer survival in melanoma patients treated with immune checkpoint inhibitors. Although its incremental predictive value was modest, allostatic load is a feasible, pre-treatment marker of host physiological vulnerability derived from routine clinical data. Further prospective studies are needed to validate these findings and clarify clinical utility.

## Figures and Tables

**Table 1:** Demographic and clinical characteristics of melanoma patients treated with immunotherapy (N = 399).

	N (%)	AL (Mean (SD))	*p* Value
Total	399 (100%)	4.43 (2.13)	
Age of first drug			
≤64	196 (49.12%)	4.21(2.12)	
>64	195 (48.87%)	4.64 (2.09)	0.04
Gender			
Male	256 (64.16%)	4.57 (2.19)	
Female	143 (35.84%)	4.15 (1.97)	0.05
Race			
White	386 (96.74%)	4.43 (2.13)	
Black	4 (1.00%)	4.50 (1.29)	
Other	4 (1.00%)	2.50 (1.29)	0.19
Stage			
III	42 (10.55%)	4.23 (1.46)	
IV	356 (89.22%)	4.45 (2.19)	0.68
Brain metastases			
No	288 (33.58%)	4.37 (2.04)	
Yes	111 (15.54%)	4.55 (2.32)	0.48
Mutation			
*BRAF*−	196 (49.12%)	4.43 (2.20)	
*BRAF+*	131 (32.83%)	4.22 (2.04)	
Other	72 (18.05%)	4.76 (2.04)	0.22
Therapy Drug			
CTLA-4 + PD-1	196 (49.12%)	4.31 (2.01)	
PD-1	203 (50.88%)	4.53 (2.23)	0.31
Treatment toxicity			
None	224 (56.14%)	4.37 (2.25)	
Grade 1	116 (29.07%)	4.23 (1.85)	
Grade 2	23 (5.76%)	5.35 (2.06)	
Grade 3	9 (2.26%)	5.44 (2.19)	
Grade 4	2 (0.50%)	5.50 (0.71)	0.10
Adjuvant Therapy			
No	339 (84.96%)	4.46 (2.11)	
Yes	58 (14.54%)	4.07 (2.05)	0.19
Response			
CR	57 (17.53%)	4.56 (1.98)	
PR	97 (29.84%)	4.39 (1.92)	
SD	67 (20.62%)	4.91 (2.04)	
PD	104 (32.00%)	4.20 (2.30)	0.18
Progression			
No	218 (54.64%)	4.34 (2.06)	
Yes	150 (37.59%)	4.51 (2.18)	0.46
Death			
Yes	212 (53.13%)	4.03 (1.93)	
No	187 (46.87%)	4.87 (2.24)	<0.01

**Table 2:** Association between pre-treatment AL and treatment toxicity grade among melanoma patients treated with immunotherapy.

	Crude Model		Model 1		Model 2		Model 3	
Toxicity Grade	ORs (95% CI)	*p* Value	ORs (95% CI)	*p* Value	ORs (95% CI)	*p* Value	ORs (95% CI)	*p* Value
No	Ref		Ref		Ref		Ref	
Grade 1	0.95 (0.85, 1.06)	0.32	0.98 (0.87, 1.20)	0.65	0.97 (0.86, 1.09)	0.58	0.98 (0.86, 1.10)	0.74
Grage 2~4	1.21 (1.03, 1.43)	0.02	1.29 (1.09, 1.53)	<0.01	1.27 (1.07, 1.52)	<0.01	1.30 (1.08, 1.57)	<0.01

Model 1: adjusted with age and gender. Model 2: adjusted with age, gender, stage, brain metastases, and *BRAF* mutation. Model 3: adjusted with age, gender, stage, *BRAF* mutation, brain metastases, regimen, and adjuvant therapy.

**Table 3:** Association between pre-treatment AL and treatment response among melanoma patients treated with immunotherapy.

	Crude Model		Model 1		Model 2		Model 3	
	ORs (95% CI)	*p* Value	ORs (95% CI)	*p* Value	ORs (95% CI)	*p* Value	ORs (95% CI)	*p* Value
	All patients (N = 399)							
Response								
CR	Ref		Ref		Ref		Ref	
PR	0.96 (0.82, 1.12)	0.57	0.99 (0.85, 1.14)	0.86	0.99 (0.85, 1.15)	0.87	1.01 (0.85, 1.20)	0.88
SD	1.08 (0.91, 1.27)	0.38	1.09 (0.91, 1.30)	0.33	1.08 (0.91, 1.29)	0.36	1.09 (0.91, 1.30)	0.35
PD	0.92 (0.79, 1.07)	0.29	0.93 (0.80, 1.09)	0.29	0.92 (0.78, 1.08)	0.31	0.94 (0.79, 1.12)	0.47
Response								
CR/PR	Ref		Ref		Ref		Ref	
SD/PD	1.01 (0.91, 1.12)	0.91	1.00 (0.90, 1.11)	0.99	0.99 (0.89, 1.11)	0.90	0.99 (0.89, 1.11)	0.91
	No toxicity (N = 224)							
Response								
CR	Ref		Ref		Ref		Ref	
PR	1.01 (0.83, 1.24)	0.91	1.06 (0.84, 1.33)	0.61	1.05 (0.83, 1.33)	0.68	1.09 (0.84, 1.41)	0.53
SD	1.01 (0.80, 1.27)	0.93	1.03 (0.81, 1.31)	0.79	1.02 (0.80, 1.31)	0.86	1.02 (0.78, 1.34)	0.89
PD	0.89 (0.72, 1.11)	0.31	0.92 (0.74, 1.15)	0.47	0.89 (0.71, 1.11)	0.29	0.91 (0.72,1.16)	0.44
Response								
CR/PR	Ref		Ref		Ref		Ref	
SD/PD	0.92 (0.80, 1.05)	0.23	0.92 (0.80, 1.06)	0.26	0.91 (0.79, 1.05)	0.20	0.90 (0.78, 1.04)	0.16
	Toxicity (N = 150)							
Response								
CR	Ref		Ref		Ref		Ref	
PR	0.91 (0.70, 1.18)	0.48	0.92 (0.71, 1.19)	0.52	0.88 (0.68, 1.13)	0.33	0.88 (0.67, 1.16)	0.37
SD	1.29 (0.97, 1.70)	0.08	1.29 (0.97, 1.72)	0.08	1.31 (0.97, 1.77)	0.08	1.38 (1.01, 1.89)	0.04
PD	1.04 (0.78, 1.39)	0.78	1.05 (0.78, 1.41)	0.75	1.00 (0.75, 1.34)	0.97	1.01 (0.74, 1.38)	0.93
Response-Recode								
CR/PR	Ref		Ref		Ref		Ref	
SD/PD	1.22 (1.01, 1.49)	0.04	1.23 (1.01, 1.50)	0.04	1.22 (0.99, 1.50)	0.06	1.24 (1.01, 1.54)	0.04

Model 1: adjusted with age and gender. Model 2: adjusted with age, gender, toxicity (all patients only), stage, brain metastases, and *BRAF* mutation. Model 3: adjusted with age, gender, toxicity (all patients only), stage, *BRAF* mutation, brain metastases, regimen, and adjuvant therapy.

**Table 4:** Association between pre-treatment AL with progression and overall survival among melanoma patients treated with immunotherapy.

	Crude	Model 1	Model 2	Model 3
	HRs (95% CI)	HRs (95% CI)	HRs (95% CI)	HRs (95% CI)
	Progression			
All patients	1.02 (0.95, 1.10)	1.04 (0.96, 1.12)	1.07 (0.97, 1.18)	1.12 (1.01, 1.24)
No toxicity	0.98 (0.89, 1.07)	0.99 (0.90, 1.09)	0.99 (0.89, 1.09)	1.00 (0.91, 1.11)
Toxicity	1.15 (0.98, 1.33)	1.15 (0.99, 1.34)	1.13 (0.97, 1.32)	1.14 (0.98, 1.33)
	Overall survival			
All patients	1.15 (1.08, 1.23)	1.13 (1.05, 1.21)	1.12 (1.04, 1.21)	1.26 (1.14, 1.39)
No toxicity	1.13 (1.04, 1.21)	1.11 (1.03, 1.21)	1.12 (1.03, 1.22)	1.23 (1.10, 1.37)
Toxicity	1.29 (1.11, 1.50)	1.27 (1.09, 1.48)	1.16 (0.98, 1.38)	1.19 (0.99, 1.42)

Model 1: adjusted with age and gender. Model 2: adjusted with age, gender, toxicity (all patients only), progression status (overall survival only), stage, brain metastases, and *BRAF* mutation. Model 3: adjusted with age, gender, toxicity (all patients only), progression status (overall survival only), stage, *BRAF* mutation, brain metastases, regimen, and adjuvant Therapy.

## Data Availability

The data generated in this study are available within the article and its Supplementary Data Files.

## Supplementary Materials

The following supporting information can be downloaded at: https://www.mdpi.com/article/10.3390/cancers18040606/s1, Table S1: Distribution and high-risk thresholds of individual biomarkers for AL; Table S2: Sensitivity analysis using alternative AL construction; Table S3: Levels of pre-treatment AL by affected organ systems among melanoma patients treated with immunotherapy; Table S4: Levels of pre-treatment AL by clinical severity of affected organ systems among melanoma patients treated with immunotherapy; Table S5: Stratified analysis to assess the relationship between pre-treatment AL with overall survival among melanoma patients treated with immunotherapy and experiencing toxicity.
