# Supplementary material for: Allostatic Load Predicts Immune-Related Toxicity and Survival in Melanoma Patients Receiving Immune Checkpoint Inhibitors
Source: Cancers (Basel). 2026 Feb 12;18(4):606. doi: 10.3390/cancers18040606 (PMC12938773; doi:10.3390/cancers18040606)
Supplement: Supplementary file 1 [file cancers-18-00606-s001.zip › cancers-4121542-supplementary.pdf]

cancers

MDPI

Table S1. Distribution and high-risk thresholds of individual biomarkers for AL.

		Initial (N=399)			After Imputation (N=399)	
	Cutoff Value	at Risk	% at Risk	% missing	at Risk	% at Risk
SBP-mmHg	≥140	148	37.09	1.50	150	37.59
DBP-mmHg	≥90	26	6.52	1.50	28	7.02
Pulse	>=100	38	9.52	1.50	38	9.52
BMI	>=25	269	67.42	4.01	279	69.92
Fast-gluc-mg/dl	>=126	121	30.33	1.50	123	30.83
Albumin-g/dl	>5.5 or <3.5	78	19.55	2.01	82	20.55
Creatinine-mg/dl	>1.1 or <0.6	89	22.31	1.50	92	23.06
eGFR-ml/min	<90	216	54.14	5.51	228	57.14
WBC-k/ul	>11 or <4.5	86	21.55	2.01	88	22.06
BNU-mg/dl	>20 or <7	127	31.83	1.50	129	32.33
ALP-IU/l	>147 or <44	59	14.79	1.50	60	15.04
LDL-mg/dl	>=130	65	20.01	18.80	83	20.80
Total Chol-mg/dl	>=240	41	12.06	17.54	47	11.78
HDL-mg/dl	<=50	194	54.96	11.28	235	58.90
Trig-mg/dl	>=150	118	34.81	15.04	148	37.09

Table S2. Sensitivity analyses using alternative AL construction.

	Quartile AL	
Toxicity Grade	ORs (95% CI)	P value
No	Ref	
Grade 1	0.88(0.80,1.08)	0.32
Grage2~4	1.13(1.01,1.32)	0.02
	Core multisystem-AL (8 components)	
Toxicity Grade		
No	Ref	
Grade 1	0.97(0.81,1.15)	0.70
Grage2~4	1.46(1.10,1.93)	0.009

Table S3. Levels of pre-treatment AL by affected organ systems among melanoma patients treated with immunotherapy.

**Affected organ system**	**Toxicity event (%)**	**Mean AL (SD)**
Gastrointestinal system	48 (32.2%)	4.73 (1.94)
Hepatobiliary and pancreatic system	35 (23.5%)	4.44 (1.99)
Endocrine system	31 (20.8%)	4.48 (2.22)
Dermatologic (skin and mucosal) system	23 (15.4%)	4.83 (1.40)
Pulmonary (respiratory) system	19 (12.8%)	4.16 (1.89)
Other or unclassified	10 (6.7%)	4.50 (2.22)
Cardiovascular system	6 (4%)	6.17 (2.14)
Ocular (eye) system	6 (4%)	5.33 (2.07)
Hematologic (blood) system	4 2.7%)	5.25 (1.71)
Neurologic and neuromuscular system	4 (2.7%)	6.25 (2.75)
Constitutional symptoms (general)	2 (1.3%)	6.50 (2.12)
Renal (kidney) system	2 (1.3%)	3.50 (0.71)
Secondary malignancy	1 (0.7%)	6.00

Table S4. Levels of pre-treatment AL by clinical severity of affected organ systems among melanoma patients treated with immunotherapy.

AE severity level	AE Event (%)	Mean AL (SD)	P value
Severe	29 (19.5 %)	5.59 (2.27)	<0.01
Moderate	87 (58.4 %)	4.60 (1.93)	
Mild	56 (37.6 %)	4.60 (1.90)	
Minimal	19 (12.8 %)	5.44 (2.13)	

Table S5. Stratified analysis to assess the relationship between pre-treatment AL with overall survival among melanoma patients treated with immunotherapy and experiencing toxicity.

	HR (95% CI)	P value
	By progression status	
No (N=84)	1.07 (0.78, 1.48)	0.67
Yes (N=49)	**1.26 (1.01, 1.58)**	**0.04**
	By *BRAF* mutation status	
No (N=63)	1.21 (0.95, 1.54)	0.10
Yes (N=56)	1.03 (0.68, 1.58)	0.90

Adjusted with age, gender, progression status, stage, *BRAF* mutation, brain metastases, regimen, and adjuvant Therapy a.

https://doi.org/10.3390/cancers18040606
